# The Application of Deep Learning on CBCT in Dentistry

**DOI:** 10.3390/diagnostics13122056

**Published:** 2023-06-14

**Authors:** Wenjie Fan, Jiaqi Zhang, Nan Wang, Jia Li, Li Hu

**Affiliations:** 1 Department of Stomatology, Union Hospital, Tongji Medical College, Huazhong University of Science and Technology, Wuhan 430022, China; 2 School of Stomatology, Tongji Medical College, Huazhong University of Science and Technology, Wuhan 430030, China

**Keywords:** cone beam computed tomography, deep learning, medical decision-making aids, segmentation, diagnosis

## Abstract

Cone beam computed tomography (CBCT) has become an essential tool in modern dentistry, allowing dentists to analyze the relationship between teeth and the surrounding tissues. However, traditional manual analysis can be time-consuming and its accuracy depends on the user’s proficiency. To address these limitations, deep learning (DL) systems have been integrated into CBCT analysis to improve accuracy and efficiency. Numerous DL models have been developed for tasks such as automatic diagnosis, segmentation, classification of teeth, inferior alveolar nerve, bone, airway, and preoperative planning. All research articles summarized were from Pubmed, IEEE, Google Scholar, and Web of Science up to December 2022. Many studies have demonstrated that the application of deep learning technology in CBCT examination in dentistry has achieved significant progress, and its accuracy in radiology image analysis has reached the level of clinicians. However, in some fields, its accuracy still needs to be improved. Furthermore, ethical issues and CBCT device differences may prohibit its extensive use. DL models have the potential to be used clinically as medical decision-making aids. The combination of DL and CBCT can highly reduce the workload of image reading. This review provides an up-to-date overview of the current applications of DL on CBCT images in dentistry, highlighting its potential and suggesting directions for future research.

## 1. Introduction

**Figure 1:**
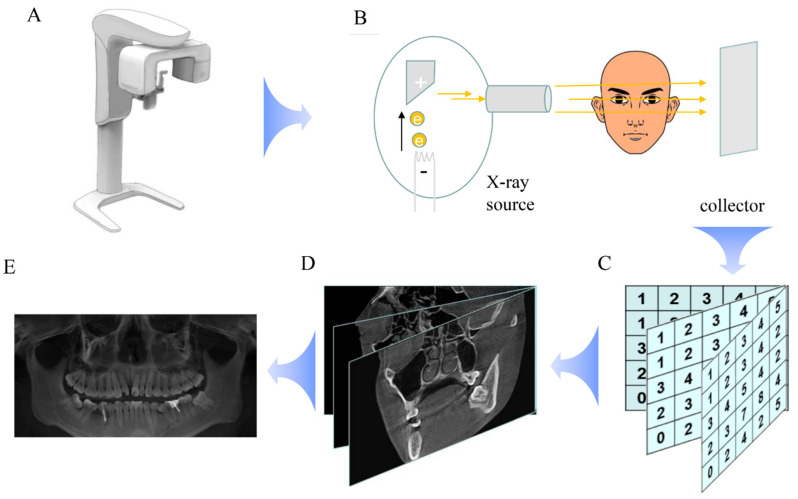
The main principles of CBCT. CBCT (**A**) consists of an X-ray source and collector. The X-ray source produces X-rays which penetrate the head and are collected by the collector (**B**). The collector translates the X-rays into digital signals (**C**). Those numbers are used to calculate the values of every point of the head by the Lambert-Beer Law and Radon transform (**D**). Finally, all values are summarized and synthesized into CBCT images (**E**).

Before the 1990s, dental X-rays were only applied in 2D images, such as panoramic radiographs [1]. In 1998, P. Mozzo invented a new computed tomography (CT), the first CBCT, which had the advantage of low X-ray doses and could be applied well for dento-maxillofacial images. Its most important advantage was the 3D image [2]. As per his prediction, CBCT has become an indispensable tool in modern oral medicine, fulfilling its promise as a non-invasive imaging technique that enables the visualization of both hard and soft tissues within the maxillofacial region. The CBCT apparatus is composed of an X-ray source and collector, which function similarly to traditional CT scanners. At the X-ray source, electrons produced in the cathode strike the anode, with most of the energy being transformed into heat, while only a few are converted into X-rays via the Bremsstrahlung effect. Meanwhile, collectors receive X-rays across the patient’s head and translate the photons into electrical signals. By revolving around the mandibular region, the X-ray tube and collector can obtain multiple slices of the head and related 2D data. This information is then processed to construct 3D models [3]. The calculation principle underlying this process involves the Lambert–Beer law and the Radon transform. The Lambert–Beer law states that, when X-rays penetrate an object, their strength decreases, such that it is possible to estimate the density of the tissue through the attenuation of the X-ray beam [4]. On the other hand, the Radon transform is employed to calculate the data of each point in the 3D field based on the original 2D data and slices [5]. Such mathematical operations enable the reconstruction of the scanned anatomy in 3D space, providing accurate visual representations of the internal structures of the maxillofacial region (Figure 1). Overall, CBCT has revolutionized the field of oral medicine by improving diagnostic accuracy and treatment outcomes while minimizing patient radiation exposure and invasiveness.

CBCT takes nearly half a minute to acquire the image of a patient, so breathing and other actions can induce motion artifacts [6]. This shortcoming limits its usage in children and some patients who cannot remain still during the examination. Additionally, the presence of metal can lead to metal artifacts during scanning. New algorithms have been designed to reduce these artifacts and achieved good results [7], but they still cannot be eliminated. Another shortcoming is the low quality of soft tissue caused by low X-ray doses and a spatially dependent bias, which could be addressed by enhancing the image contrast and density quantification [8].

In clinical practice, CBCT is imperative. Compared to a panoramic radiograph, CBCT contains more information. Through CBCT images, doctors can identify the boundaries of caries, periapical disease, bone disease, impacted tooth, sinus, and inferior alveolar nerve easily [2]. However, it comes with the trade-off of higher radiation exposure compared to traditional panoramic and bitewing radiographs. In recent CBCT image scanning software, there are many functions, for example, 3D scanning and reconstruction, which a panoramic radiograph does not have. The 3D nature of CBCT can help doctors to know the region of disease accurately. However, it is time-consuming for doctors to identify every landmark and measure parameters on CBCT images. Moreover, it takes a long time for new doctors to become proficient in CBCT landmarks. The development and application of automation will help to solve these problems. Therefore, we present a summary of the application of automation, hoping to provide new ideas for future research and promote the development of CBCT image reading automation.

## 2. Deep Learning

DL is a subset of machine learning (ML), which belongs to artificial intelligence (AI) [9]. ML allows manual feature extraction which can be used to predict some special data [10]. Deep learning is also called end-to-end ML, because it enables the entire process to map from original input images to the final classification, eliminating the need for human intervention [11].

Deep learning algorithms contain various types of neural networks, such as convolutional neural networks (CNNs), k-nearest neighbors (KNN), recurrent neural networks (RNNs), and others. These networks are designed to simulate the behavior of nerve cells in the brain. They receive input data from many sources, which is processed by nodes within the network to generate output results. In the early days, these algorithms were relatively simple input–output models, but they have since evolved into complex and sophisticated systems that can handle large amounts of data and perform advanced tasks such as image recognition, natural language processing, and predictive modeling [9].

**Figure 2:**
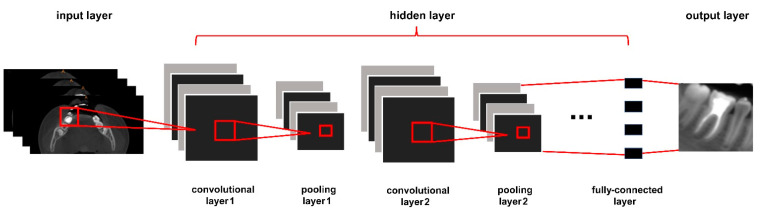
The structure of a CNN.

**CNNs**. In 2006, professor Geoffrey Hinton and his student described an effective way to initialize the weights that worked well [12]. This work brought neural networks to the forefront of research again. Nowadays, CNNs are the most widely used neural networks in medical image segmentation and analysis. CNNs contain an input layer, an output layer, and hidden layers. Hidden layers contain many pooling layers, convolutional layers, and fully connected layers, as shown in Figure 2 [13,14]. Convolutional filters can learn image features and extract hierarchical features. The pooling layer is used for averaging all acquired features and relating them to neighboring pixels [15]. U-Net is one of the most important frameworks of CNNs [16]. It is also widely used in medical image segmentation.

**KNN**. KNN is a simple algorithm which is mostly used to classify a data point based on how its neighbors are classified [17].

**RNNs**. The characteristic of an RNN is that the neurons in the hidden layer are connected. The time-related input information in the sliding window can be transmitted sequentially, and the temporal correlation between distant events in the temporal dimension can be considered [18]. RNNs perform well in automatic speech recognition applications.

Medical imaging is one of the largest and most promising applications of deep learning in healthcare. At present, with the development of society, imaging examination is more and more common, and the social demand for radiologists and automated diagnosis is also gradually increasing [19]. Deep learning provides a way to solve these problems [20]. Deep learning has been studied in many medical fields, such as ophthalmology, respiratory, orthopedics, etc. [21,22,23]. In recent years, the application of DL in dentistry has also increased fast and DL is the most popular AI method applied in dentistry [10]. In many dental fields, the accuracy of DL is similar to, or even better than, manual work [24].

## 3. The Application of Deep Learning in CBCT

In clinical practice, the application of DL in CBCT can help the doctor in their diagnosis. It includes an array of pre-processing, segmentation, and classification techniques that form an automated dental identification system, facilitating the work of dentists [25]. It can also narrow the gap between old and new doctors’ abilities to read images, and alleviate the gap between imaging diagnoses in rich and poor areas.

However, there are many challenges in this field, such as poor image quality, irregular object shape, intensity variation in X-rays, proper selection of method, limitations of the capture device, label and annotation reliability, and a lack of available datasets [25,26]. In addition to these technical and data factors, the main issue is ethical [27]. Deep learning cannot take responsibility for patients when a diagnosis goes wrong, which may mean that it can only be used as auxiliary medical equipment.

**Figure 3:**
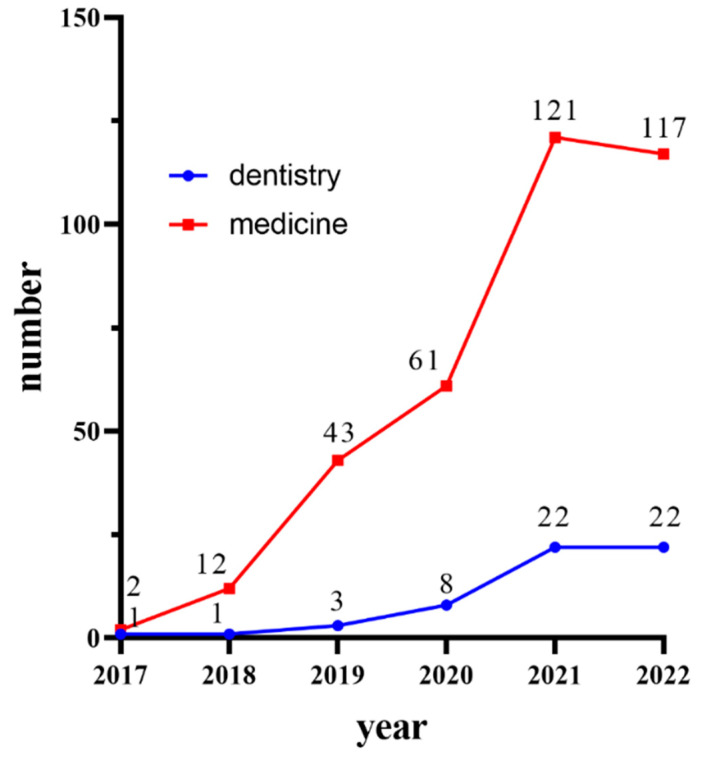
The statistics of DL studies on CBCT in medicine and dentistry from 2017 to 2022.

In recent years, the application of DL on CBCT has developed rapidly. We searched the literature on Pubmed, IEEE, Google Scholar, and Web of Science up to December 2022. The combinations of search terms were constructed from “artificial intelligence”, “AI”, “deep learning”, “DL”, “convolution neural network”, “automatic”, “computer-assisted diagnosis”, “Cone beam CT”, and “CBCT”. We obtained 356 articles about DL application in medicine, but some of them did not belong to dentistry. We only wanted to summarize the application of DL on CBCT in dentistry. The application of image quality improvement, tumor radiology therapy, and other fields were not considered. Finally, we found 54 articles about the clinical application of DL in CBCT (Figure 3), which showed a rapidly developing trend. We summarized the data of the studies and wrote this narrative review.

Most of the studies calculated true positive (TP), true negative (TN), false positive (FP) and false negative (FN). TP represents a region which was supposed to be segmented and was correctly segmented; FN refers to a region which should have been but was not segmented; FP is a region which was segmented but was not supposed to be segmented; and TN represents a region which was not supposed to be segmented and was not segmented. In the tables below, we have summarized the accuracy, precision, recall or sensitivity, Dice similarity coefficient (DSC), intersection over union (IoU), F1 score, and 95% Hausdorff distance (HD) for the studies included in this review.

Accuracy: The rate of correct findings in relation to all of the observed findings.

Accuracy = (TP + TN)/(TP + TN + FP + FN)

Precision: The percentage of the accurately segmented area out of the completely segmented area.

Precision = TP/(TP + FP)

Recall or sensitivity: The percentage of the regions that were perfectly detected.

Recall = TP/(TP + FN) = Sensitivity

F1 score: The harmonic average of precision and recall.

F1 score = 2 × precision × recall/(precision + recall)

DSC: The score of how much the segmented area was similar to the ground truth.

DSC = 2TP/(FP + 2TP + FN)

IoU: The amount of overlap between the predicted segmentation and the ground truth.

IoU = TP/(TP + FP + FN)

95% HD: Provides the 95th percentile of the maximal distance between the boundaries of the automatic segmentation and the ground truth.

$$P_{95}\left(\underset{g\in G}{min}{\left\|p-g\right\|}_{2}\cup^{\;}\underset{p\in P}{min}{\left\|g-p\right\|}_{2}\right)$$

In this narrative review, we have provided a brief overview of some of the technical details of deep learning (DL), which is a well-established field and extensively covered in many other articles. However, our primary focus is on the emerging applications of DL in dentistry, particularly with respect to cone beam computed tomography (CBCT). By reviewing the current literature on the topic, we aim to provide insights and guidance for future research on DL applied to CBCT in the context of dentistry. According to the different organizational areas and common applications, we have divided them into eight categories. They are the upper airway, inferior alveolar nerve and the third molar, bone-related disease, tooth segmentation, temporal-mandibular joint (TMJ) and sinus disease, dental implant, and landmark localization.

### 3.1. The Application of Deep Learning in CBCT in Segmentation of the Upper Airway

Upper airway reconstruction is essential in the diagnosis and treatment of diseases such as obstructive sleep apnea-hypopnea syndrome (OSAHS) and adenoidal hypertrophy. The use of deep learning with CBCT has enormous potential to improve these fields. By segmenting the upper airway, the volume can be calculated and used for assessing upper airway obstruction. These applications are mostly semi-automatic or automatic, which can save time for doctors. Many studies have reported high accuracy and specificity, with 3D U-Net achieving the highest accuracy. However, most studies did not report the algorithm’s runtime, except for one study. As such, there is still plenty of room for improvement in terms of speed. Nonetheless, the application of deep learning in these areas shows great promise for improving patient outcomes and reducing the workload of medical professionals.

The 3D U-Net neural network is the most widely studied neural network in upper airway segmentation. It was used to detect and segment airway space and help diagnose OSAHS. The best accuracy for pharyngeal airway segmentation can reach 0.97 ± 0.01 and the Dice score is 0.97 ± 0.02 [28]. Only one study has reported the time taken for analysis, reporting that it took nearly 10 min to analyze each sample. However, this may be an overestimation of the time, because it not only contained pharyngeal airway segmentation, but also contained computational fluid dynamics calculation and OSAHS assessment [29]. In some trials, doctors have assessed that the accuracy of 3D U-Net was ready for clinical assistance in OSAHS diagnosis [30].

CNNs are the second most studied algorithm and also perform well. Leonardi et al. describe a CNN method to segment the sinonasal cavity and pharyngeal airway on CBCT images. Furthermore, there was no difference between the manual group and the CNN group [31]. Ulaş Öz also chose CNN to segment the upper airway and calculate its volume. The mean accuracy was 96.1% and the Dice score reached 91.9% [32].

Only one study used a regression neural network as the main algorithm. Their test showed that this model was as accurate as manual segmentation [33].

The existing DL models on upper airway segmentation have been shown in Table 1.

### 3.2. The Application of Deep Learning in CBCT in Segmentation of the Inferior Alveolar Nerve

Inferior alveolar nerve injury, which can cause temperature, pain, touch, and pressure sensation disorder in the mandibular parts, is one of the commonest complications of implant surgery, molar extraction, and orthognathic surgery. Compared to panoramic radiography, CBCT has a higher predictive value before surgery [34]. In clinical practice, detection and segmentation of the IAN on CBCT images is a necessary task prior to implant surgery, molar extraction, and orthognathic surgery. However, this process is time-consuming and requires skilled manual labor. Recently, deep learning has shown promising results in automating this task, thus significantly reducing the time required for this necessary step in clinical diagnosis. However, the accuracy in this field is acceptable, but the precision and DSC still need to be improved, which can ultimately lead to improved patient outcomes in dentistry.

CNNs are the most used method in this field. Cipriano et al. described a public and complete method of detecting IAN with CNN and its Dice score was 0.69 [35]. They did not calculate the accuracy. Many other researchers have described some high-quality methods of detecting IAN with CNNs on CBCT images, but some of their data were not available. Their best accuracy could even reach 0.99 [36,37,38]. A new study compared the difference between specialist doctors and DL based on CNNs using a large sample of people who came from different nations and five kinds of CBCT devices. It verified that DL had lower variability than the interobserver variability between the radiologists [39]. In addition to detecting IAN alone, CNNs have also been used to detect the relationship between IAN and the third molar by Pierre Lahoud and Mu-Qing Liu [40,41]. Their studies all reached high accuracy. The mean DSC in Liu’s method could reach 0.9248. The method found by Lahoud could detect IAN in nearly 21.26 s. Furthermore, the continuity-aware contextual network (Canal-Net) was constructed based on 3D U-Net with bidirectional convolutional long short-term memory (ConvLSTM) under a multi-task learning framework. Conventional deep learning algorithms (2D U-Net, SegNet, 3D U-Net, MPL 3D U-Net, ConvLSTM 3D U-Net) and Canal-Net were assessed in the study. Canal-Net performed better and had clearer boundary detection. It also achieved a higher accuracy and Dice score compared to the other algorithms [42].

The existing DL models on inferior alveolar nerve have been shown in Table 2.

### 3.3. The Application of Deep Learning in CBCT in Bone-Related Disease

CT has an advantage in bone imaging and CBCT inherits this advantage as well. Furthermore, CBCT produces less radiation and saves cost. So, compared to CT, CBCT has a huge advantage in maxillofacial bone disease diagnosis. Some researchers also agreed that panoramic radiographs are insufficient in complicated facial fracture diagnosis [44]. Therefore, the research and applications of DL in CBCT are imperative in maxillofacial bone disease.

CNNs have been used in jaw bone transmissive lesion detection on CBCT images, and its overall accuracy can reach nearly 80% [45]. In this study, the jaw bone transmissive lesions contained ameloblastoma, periapical cysts, dentigerous cysts, and keratocystic odontogenic tumors (KCOT). However, in this study, CNNs could not classify which type of disease the lesion belonged to. There are other scientists who have studied the computer-aided CBCT diagnosis system. It can classify periapical cysts and keratocystic odontogenic tumor lesions. However, the authors did not clarify the classification of their method [46].

Recently, there have been many applications of DL in bone lesion detection on CT images [47]. DL can also be used to diagnose bone tumors, bone cysts, fractures, and jaw deformities.

The existing DL models on bone-related disease have been shown in Table 3.

### 3.4. The Application of Deep Learning in CBCT in Tooth Segmentation and Endodontics

Tooth segmentation has been the focus of much research in the application of DL in dentistry. It can be divided into two types: global segmentation and partial segmentation. Global segmentation is useful for generating tooth charts and orthodontic plans. In particular, DL and CBCT-based global segmentation techniques can provide more comprehensive dental information compared to recent oral scans, which only show the position and axis of the crown but not the root. This approach can save time in the diagnosis and treatment planning process for orthodontic patients. On the other hand, partial segmentation techniques are applied to aid in the diagnosis of dental diseases such as periapical disease, pulpitis, and root fractures. These techniques involve the identification and localization of specific regions of interest within the tooth structure, which can help clinicians make more informed decisions about appropriate treatment options.

In tooth segmentation, Kang Cheol Kim et al. described an automatic tooth segmentation method based on CBCT imaging, but they did not say which algorithm was used. They first changed the 3D image into a 2D image and identified 2D teeth. Then, loose and tight regions of interest (ROIs) were captured. Finally, the accurate 3D tooth was segmented by loose and tight ROIs. The accuracy could reach 93.35% and the Dice score reached 94.79% [48]. There are also many studies about tooth segmentation and identification, and they all obtain good results [49,50,51,52,53]. Most of their methods used CNNs or were based on CNNs. There are few studies on U-net. Some traditional U-Net methods (2Da U-Net, 2Dc U-Net, 2Ds U-Net, 2.5Da U-Net, and 3D U-Net) were compared with upgraded versions of U-Net (2.5Dv U-Net, 3.5Dv5 U-Net, 3.5Dv4 U-Net, and 3.5Dv3 U-Net) which were obtained using majority voting in tooth segmentation. The best performing method was 3.5Dv5 U-Net and the DSC reached 0.922 [54].

In periapical disease, DL performs well. A CNN method was studied to detect periapical pathosis and calculate their volumes on CBCT images. The result showed no difference between DL and manual segmentation and the accuracy could reach 92.8% [55]. Setzer et al. used a deep learning method based on U-Net to segment periapical lesions on CBCT images. The accuracy of lesion detection was 0.93 and the DSC for all true lesions was 0.67 [56]. It verified that the accuracy of DL can reach the quality of manual working. However, the DSC still needs to be improved.

In root canal system detection, Zhang Jian used 3D U-Net to recognize root canals. They solved the class imbalance problem and developed the ability to segment using the CLAHE algorithm and a combination loss based on dice loss [57]. U-Net can be used to detect the C-shaped root canal of the second molar and unobturated mesial buccal 2 (MB2) canals on endodontically obturated maxillary molars on CBCT images [58,59]. A cGAN model was used to segment different tooth parts, and the segmentation effect was ideal [60]. Deep learning methods can also be used in combination. In tooth pulp segmentation, a two-step method was reported. First, a region proposal network (RPN) with a feature pyramid network (FPN) method was applied to detect single-rooted or multirooted teeth. Second, they used U-Net models to segment the pulp. This method can obtain accurate tooth and pulp cavity segmentation [61].

Many deep learning methods have been combined in root segmentation. Li et al. described a root segmentation method based on U-Net with AGs, and RNN was applied for extracting the intra-slice and inter-slice contexts. The accuracy was higher than 90% [62]. In vertical root fracture diagnosis, Ying Chen and his team accessed three deep learning networks (ResNet50, VGG19, and DenseNet169) with or without previous manual detection. In the manual group the accuracy of deep learning could reach 97.8% and in the automatic group was 91.4%. It showed that deep learning has huge potential in the assistance of diagnosis [63].

The existing DL models on tooth segmentation have been shown in Table 4.

### 3.5. The Application of Deep Learning in CBCT in TMJ and Sinus Disease

In TMJ and sinus disease detection, CBCT can show its 3D advantage clearly. The panoramic radiograph can only show whether there is disorder, but CBCT can also show where the disorder is.

U-Net was used to segment the mandibular ramus and condyles in CBCT images; the average accuracy was near 0.99 [65]. Classification of temporomandibular joint osteoarthritis (OA) can be identified by a web-based system based on a neural network and shape variation analyzer (SVA) [66,67].

Except for OA and the morphology of condyles, CBCT can also show the joint space, effusion, and mandibular fossa which also can provide evidence for TMJDS diagnosis. However, there is no study of the application of DL in temporal-mandibular joint CBCT diagnosis.

CNNs have been used to diagnose sinusitis. It was demonstrated that the accuracy of CBCT was much higher than panoramic radiographs and the accuracy of CBCT can reach 99.7% [68]. Other scientists also performed similar research, 3D U-Net was used to segment the bone, air, and lesion of the sinus [69]. However, the algorithm for sinus lesions still needs to be improved.

The existing DL models on TMJ and sinus disease have been shown in Table 5.

### 3.6. The Application of Deep Learning in CBCT in Dental Implant

Before implant surgery, doctors always need to measure the bone density, width, and depth, and decide on the implant’s position. The integration of CBCT imaging and DL techniques can help doctors to collect and analyze those messages.

Bone density relates to the implant choice and the placing of the implant insertion. Knowing the alveolar bone density in advance can also help doctors to select the implant tool. Many kinds of DL methods have been studied. CNNs were studied to make classifications of alveolar bone density on CBCT images through a 6-month follow-up. The accuracy could reach 84.63% and 95.20% in hexagonal prism and cylindrical voxel shapes, respectively [70]. Nested-U-Net was also used, and the Dice score could reach 75% [71]. QCBCT-NET, which combines a generative adversarial network (Cycle-GAN) and U-Net, can be used to measure the mineral density of bone. It was verified that QCBCT-NET was more accurate than Cycle-GAN and U-Net used singly [72].

In addition to in relation to bone density, CNNs have also been used in other areas. Faisal Saeed chose six CNN models (AlexNet, VGG16, VGG19, ResNet50, DenseNet169, and MobileNetV3) to detect missing tooth regions. Among them, DenseNet169 achieved the best score and the accuracy could reach 89% [73]. Bayrakdar et al. used a CNN to measure bone height, bone thickness, canals, sinuses, and missing teeth. They achieved good results in premolar tooth regions in bone height measurements. However, in other measurements, the results need to be improved [74]. CNNs can also can be used to help plan the immediate implant placement. A recent end-to-end model only took 0.001 s for each CBCT image analysis [75].

After implant surgery, CNNs can help to assess implant stability. Panoramic radiograph cannot show the full bone loss or integration information around the implant, so CBCT is the best choice. Liping Wang described a multi-task CNN method that can segment implants, extract zones of interest, and classify implant stability. Its accuracy was higher than 92% and it could evaluate each implant in 3.76 s [76].

The combination of CBCT and DL can aid in the evaluation of tooth loss, alveolar bone density, height, thickness, location of the inferior alveolar nerve, and other conditions in the area of tooth loss. Such information provides a basis for doctors to evaluate the feasibility of implantation and shorten the time required for treatment planning. Additionally, postoperative stability analysis can be performed using these technologies, providing convenience for later review. These existing techniques already cover preoperative assessment and postoperative follow-up for implant surgery. As technology advances, the combination of these techniques may pave the way for the development of implant surgery robots in the near future.

The existing DL models on implant have been shown in Table 6.

### 3.7. The Application of Deep Learning in CBCT in Landmark Localization

Craniomaxillofacial (CMF) landmark localization is critical in surgical navigation systems, as the accuracy of landmark localization directly impacts surgical precision. This field presents challenges for deep learning due to the presence of deformities and traumatic defects. However, the application of deep learning techniques can save time for doctors and assist in clinical planning, as accurate data enables more precise surgical plans. Overall, while challenging, deep learning showed good results in CMF landmark localization.

Neslisah Torosdagli et al. proposed a three-step deep learning method to segment the anatomy and make automatic landmarks. In the first step, they constructed a new neural network to segment the image, which decreases the complex post-processing. In the second step, they formulated the landmark localization problem for automatic landmarks. In the third step, they used a long short-term memory network to identify the landmark. Their method showed very good results [77].

Shen Dinggang and his team performed a lot of work in this field. They described a multi-task deep neural network that can use anatomical dependencies between landmarks to realize large-scale landmarks on CBCT images [78]. Shen’s team also invented a two-step method including U-Net and a graph convolution network to identify 60 CMF landmarks. The average detection error was 1.47 mm [79]. Later, they invented another two-step method involving 3D faster R-CNN and 3D MS-UNet to detect 18 CMF landmarks. They first made a cause prediction of landmark location and then redefined it via heatmap regression. It can reach state-of-the-art accuracy of 0.89 ± 0.64 mm in an average time of 26.2 s per volume [80]. Their team also used 3D Mask R-CNN to identify 105 CMF landmarks on patients with varying non-syndromic jaw deformities on CBCT images. The accuracy could reach 1.38 ± 0.95 mm [81].

This technology can also be used in orthodontics analysis. Two-dimensional X-ray cephalometry and CBCT are both needed in clinical orthodontic practice today. Fortunately, the application of automatic landmark localization in CBCT has the potential to replace 2D X-ray cephalometry. Jonghun Yoon and his team used Mask R-CNN to detect 23 landmarks and calculate 13 parameters, even in a natural head position. Their algorithm was demonstrated to be able to perform as well as manual analysis in 30 s while manual analysis needed 30 min [82].

The existing DL models on landmark localization have been shown in Table 7.

## 4. Conclusions

In summary, the application of deep learning technology in CBCT examinations in dentistry has achieved significant progress: this achievement may significantly reduce the workload of dentists in clinical radiology image reading. In many dentistry fields, such as upper airway segmentation, IAN detection, and periapical pathosis detection, the accuracy of DL can reach that of dentists [33,39,55].

However, there are many problems that need to be addressed: (1) Ethical issues prohibit using deep learning as a stand-alone approach to diagnose oral diseases. Still, it can serve as an aid to clinical decision making. (2) Although the existing studies have produced promising results, there are still many areas that require improvement. For example, the accuracy and DSC of IAN segmentation are not yet satisfactory, while bone fracture and tumor detection are largely unexplored. (3) It may be difficult for a single algorithm model to achieve high-precision identification and diagnosis of oral diseases. Instead, the integration of multiple algorithms could be a trend in DL development.

In conclusion, the potential of deep learning in improving the accuracy of radiology image analysis in dental diagnosis is enormous. Nonetheless, more significant efforts and research must be conducted to improve its diagnostic capabilities for oral diseases.

## 5. Recommendations for Future Research

In addition to improving the accuracy of the existing DL algorithms, the following areas can also be paid attention to in future research: (1) Achieving compatibility across different CBCT devices is a critical challenge that needs to be addressed. (2) While ChatGPT—based on DL—has been used in medical radiology, its performance in dentistry needs to be improved through increasing the number of training samples [83]. (3) Since oral diseases are complex and diverse, a single-function algorithm model may lead to missed diagnoses of diseases. Therefore, integrating deep learning for the diagnosis of multiple diseases may be the future direction of research in this field.

## Figures and Tables

**Table 1:** The existing DL models on upper airway segmentation and their functions and performance.

Authors	DL Models	Year	Training Dataset	Validation/Test Dataset	Functions	Best Performance of DL	Time- Consuming
Jacobs et al. [28]	3D U-Net	2021	48	25	Segmentation of pharyngeal airway space	Precision: 0.97 ± 0.02 Recall: 0.98 ± 0.01 Accuracy: 1.00 ± 0.00 DSC: 0.98 ± 0.01 IoU: 0.96 ± 0.02 95HD: 0.82 ± 0.41 mm	No
Choi et al. [29]	CNN	2021	73 for segmentation 121 for OSAHS diagnose	15 for segmentation 52 for OSAHS diagnose	Segmentation of upper airway, computational fluid dynamics and OSAHS assessment	Upper airway flow characteristics Accuracy: 0.702 ± 0.048 Sensitivity: 0.893 ± 0.048 Specificity: 0.593 ± 0.053 F1 score: 0.74 ± 0.033 DSC: 0.76 ± 0.041 OSAHS diagnosis Accuracy: 0.815 ± 0.045 Sensitivity: 0.893 ± 0.048 Specificity: 0.862 ± 0.047 F1 score: 0.0876 ± 0.033	6 min
Yuan et al. [30]	CNN	2021	102	21 for validation 31 for test	Segmentation of upper airway	Precision: 0.914 Recall: 0.864 DSC: 0.927 95HD: 8.3	No
Spampinato et al. [31]	CNN	2021	20	20	Segmentation of sinonasal cavity and pharyngeal airway	DSC: 0.8387 Matching percentage: 0.8535 for tolerance 0.5 mm 0.9344 for tolerance 1.0 mm	No
Oz et al. [32]	CNN	2021	214	46 for validation 46 for test	Segmentation of upper airway	DSC: 0.919 IoU: 0.993	No
Lee et al. [33]	Regres-sion Neural Network	2021	243	72	Segmentation of upper airway	r^2^ = 0.975, *p* < 0.001	No

**Table 2:** The existing DL models on inferior alveolar nerve and their function and performance.

Authors	DL Models	Year	Training Dataset	Validation/Test Dataset	Functions	Best Performance of DL	Time- Consuming
Grana et al. [35]	CNN	2022	68	8 for validation 15 for test	IAN detection	IoU: 0.45 DSC: 0.62	No
Kaski et al. [36]	CNN	2020	128		IAN detection	Precision: 0.85 Recall: 0.64 DSC: 0.6 (roughly)	No
Song et al. [37]	CNN	2021	83	50	IAN detection	0.58 ± 0.08	86.4 ± 61.8 s
Hwang et al. [38]	3D U-Net	2020	102		IAN detection	Background accuracy: 0.999 Mandibular canal accuracy: 0.927 Global accuracy: 0.999 IoU: 0.577	No
Nalampang et al. [39]	CNN	2022	882	100 for validation 150 for test	IAN detection	Accuracy: 0.99	No
Jacobs et al. [40]	CNN	2022	166	30 for validation 39 for test	IAN detection, relationship between IAN and the third molar	Precision: 0.782 Recall: 0.792 Accuracy: 0.999 DSC: 0.774 IoU: 0.636 HD: 0.705	21.2 ± 2.79 s
Fu et al. [41]	CNN	2022	154	30 for validation 45 for test	IAN detection, relationship between IAN and the third molar	The third molar Accuracy: 0.9726 DSC: 0.9730 IoU: 0.9606 Mandibular canal Accuracy: 0.9563 DSC: 0.9248 IoU: 0.9003	6.1 ± 1.0 s for segmentation 7.4 ± 1.0 s for classifying relation
Yi et al. [42]	Canal-Net	2022	30	20 for validation 20 for test	IAN detection	Precision: 0.89 ± 0.06 Recall: 0.88 ± 0.06 DSC: 0.87 ± 0.05 Jaccard index: 0.80 ± 0.06 Mean curve distance: 0.62 ± 0.10 Volume of error: 0.10 ± 0.04 Relative volume difference: 0.14 ± 0.04	No
Shin et al. [43]	CNN	2022	400	500	IAN detection	Precision: 0.69 Recall: 0.832 DSC: 0.751 F1 score: 0.759 IoU: 0.795	No

**Table 3:** The existing DL models on bone-related disease and their functions and performance.

Authors	DL Models	Year	Training Dataset	Validation/Test Dataset	Functions	Best Performance of DL	Time- Consuming
Li et al. [45]	CNN	2021	282	71	Jaw bone lesions detection	Overall accuracy: 0.8049	No
Kayipmaz et al. [46]	CNN	2017	50		Periapical cyst and KCOT lesions classification	Accuracy: 1 F1 score: 1	No

**Table 4:** The existing DL models on tooth segmentation and their functions and performance.

Authors	DL Models	Year	Training Dataset	Validation/Test Dataset	Functions	Best Performance of DL	Time- Consuming
Jin et al. [48]	Unknown	2022	216	223	Tooth identification and segmentation	Tooth identification Precision: 0.9681 ± 0.0167 Recall: 0.9013 ± 0.0530 F1 score: 0.9335 ± 0.0254 Tooth segment Precision: 0.9595 ± 0.0200 Recall: 0.9371 ± 0.0208 DSC: 0.9479 ± 0.0134 HD: 1.66 ± 0.72 mm	No
He et al. [49]	cGAN	2020	15,750 teeth	4200 teeth	Tooth identification and segmentation	IoU Incisor: 0.89 ± 0.087 Lateral incisor: 0.92 ± 0.068 Canine: 0.90 ± 0.053 First premolar: 0.91 ± 0.032 Second premolar: 0.93 ± 0.026 First molar: 0.92 ± 0.112 Second molar: 0.90 ± 0.035	No
Jacobs et al. [50]	CNN	2021	2095 slice	328 for validation 501 for optimization	Tooth segmentation	R-AI IoU: 0.881 ± 0.036 DSC: 0.937 ± 0.02 F-AI IoU: 0.887 ± 0.032 DSC: 0.940 ± 0.018	R-AI 72 ± 33.02 s F-AI 30 ± 8.64 s
Jacobs et al. [51]	3D U-Net	2021	140	35 for validation 11 for test	Tooth identification and segmentation	Precision: 0.98 ± 0.02 IoU: 0.82 ± 0.05 Recall: 0.83 ± 0.05 DSC: 0.90 ± 0.03 95HD: 0.56 ± 0.38 mm	7 ± 1.2 h for experts 13.7 ± 1.2 s for DL
Deng et al. [52]	CNN	2022	450	104	Tooth identification and segmentation	Accuracy: 0.913 AUC: 0.997	No
Jacobs et al. [53]	CNN	2022	140	35	Tooth identification and segmentation	Accuracy of teeth detection: 0.997 Accuracy of missing teeth detection: 0.99 IoU: 0.96 95HD: 0.33	1.5 s
Ozyurek et al. [55]	CNN	2020	2800	153	Periapical pathosis detection and their volumes calculation	Detection rate: 0.928	No
Li et al. [56]	U-Net	2020	61	12	Periapical lesion, tooth, bone, material segmentation	Accuracy: 0.93 Specificity: 0.88 DSC: 0.78	No
Schwendicke et al. [58]	Xception U-Net	2021	100	35	Detect the C-shaped root canal of the second molar	DSC: 0.768 ± 0.0349 Sensitivity: 0.786 ± 0.0378	No
Mahdian et al. [59]	U-Net	2022	90	10	Unobturated mesial buccal 2 (MB2) canals on endodontically obturated maxillary molars	Accuracy: 0.9 DSC: 0.768 Sensitivity: 0.8 Specificity: 1	No
Xie et al [60]	cGAN	2021	Improved group 40 Traditional group 40		Different tooth parts segmentation	Omit, Precision, TRP, FRP, and DSC	No
Yang et al. [61]	RPN, FRN, U-Net	2021	20		Tooth and pulp segmentation	Single root tooth DSC: 0.957 ± 0.005 ASD: 0.104 ± 0.019 mm RVD: 0.049 ± 0.017 Multiroot tooth DSC: 0.962 ± 0.002 ASD: 0.137 ± 0.019 mm RVD: 0.053 ± 0.010	No
Lin et al. [62]	U-Net, AGs, RNN	2020	1160	361	Root segmentation	IoU: 0.914 DSC: 0.955 Precision: 0.958 Recall: 0.953	No
Lin et al. [63]	ResNet50, VGG19, DenseNet169	2022	839	279	Vertical root fracture diagnosis	ResNet50 Accuracy: 0.978 Sensitivity: 0.970 Specificity: 0.985 VGG19 Accuracy: 0.949 Sensitivity: 0.927 Specificity: 0.970 DenseNet169 Accuracy: 0.963 Sensitivity: 0.941 Specificity: 0.985	No
Zhao et al. [64]	3D U-Net	2021	51	17	Root canal system detection	DSC: 0.952	350 ms

**Table 5:** The existing DL models on TMJ and sinus disease and their functions and performance.

Authors	DL Models	Year	Training Dataset	Validation/Test Dataset	Functions	Best Performance of DL	Time- Consuming
Soroushmehr et al. [65]	U-Net	2021	90	19	Mandibular condyles and ramus segmentation	Sensitivity: 0.93 ± 0.06 Specificity: 0.9998 ± 0.0001 Accuracy: 0.9996 ± 0.0003 F1 score: 0.91 ± 0.03	No
Prieto et al. [66]	Web-based system based on neural network	2018	259	34	TMJ OA classification	No	No
Prieto et al. [67]	SVA	2019	259	34	TMJ OA classification	Accuracy: 0.92	No
Ozveren et al. [68]	CNN	2022	237	59	Maxillary sinusitis evaluation	Accuracy: 0.997 Sensitivity: 1 Specificity: 0.993	No
Song et al. [69]	3D U-Net	2021	70	20	Sinus lesion segmentation	DSC: 0.75~0.77 Accuracy: 0.91	1824 s for manual 855.9 s for DL

**Table 6:** The existing DL models on implant and their functions and performance.

Authors	DL Models	Year	Training Dataset	Validation/Test Dataset	Functions	Best Performance of DL	Time- Consuming
Khajeh et al. [70]	CNN	2019	620	54 for validation 43 for test	Bone density classification	Accuracy: 0.991 Precision: 0.952	76.8 ms
Lin et al. [71]	Nested-U-Net	2022	605	68	Bone density classification	Accuracy: 0.91 DSC: 0.75	No
Yi et al. [72]	QCBCT-NET	2021	200		Bone mineral density measurement	Pearson correlation coefficients: 0.92	No
Saeed et al. [73]	CNN	2022	350	100 for validation 50 for test	Missing tooth regions detection	Accuracy: 0.933 Recall: 0.91 Precision: 0.96 F1 score: 0.97	No
Shumilov et al. [74]	3D U-Net	2021	75		Bone height\thickness\canals, missing tooth, sinus measuring	Right detection Canal: 0.722 Sinuses/fossae: 0.664 Missing tooth: 0.953	No
Chen et al. [75]	CNN	2022	2920	824 for validation 400 for test	Perioperative plan	ICCs: 0.895	0.001 s for DL 64~107 s for manual work
Wang et al. [76]	CNN	2022	1000	150	Implant stability	Precision: 0.9733 Accuracy: 0.9976 IoU: 0.944 Recall: 0.9687	No

**Table 7:** The existing DL models on landmark localization and their function and performance.

Authors	DL Models	Year	Training Dataset	Validation/Test Dataset	Functions	Best Performance of DL	Time- Consuming
Bagci et al. [77]	Long short-term memory network	2019	20,480	5120	Mandible segmentation and 9 automatic landmarks	DSC: 0.9382 95HD: 5.47 IoU: 1 Sensitivity: 0.9342 Specificity: 0.9997	No
Shen et al. [78]	Multi-task dynamic transformer network	2020	no	no	64 CMF landmarks	DSC: 0.9395 ± 0.0130	No
Shen et al. [79]	U-Net, graph convolution network	2020	20	5 for validation 10 for test	60 CMF landmarks	Accuracy: 1.69 mm	1~3 min for DL
Yap et al. [80]	3D faster R-CNN, 3D MS-UNet	2021	60	60	18 CMF landmarks	Accuracy: 0.79 ± 0.62 mm	26.6 s for DL
Wang et al. [81]	3D Mask R-CNN	2022	25	25	105 CMF landmarks	Accuracy: 1.38 ± 0.95 mm	No
Yoon et al. [82]	Mask R-CNN	2022	170	30	23 CMF landmarks	mean absolute value of deviation length: 1 mm angle: <2°	25~35 min for manual 17 s for DL

## Data Availability

Data sharing is not applicable.
